# Supplementary material for: Prognostic Significance of CD8 T-cell Spatial Biomarkers in ER+ and ER− Breast Cancer
Source: medRxiv. 2025 May 28:2025.05.27.25328389. Preprint. [Version 1] doi: 10.1101/2025.05.27.25328389 (PMC12148275; doi:10.1101/2025.05.27.25328389)
Supplement: 1 [file NIHPP2025.05.27.25328389v1-supplement-1.pdf]

medRxiv preprint doi: https://doi.org/10.1101/2025.05.27.25328389; this version posted May 28, 2025. The copyright holder for this preprint
(which was not certified by peer review) is the author/funder, who has granted medRxiv a license to display the preprint in perpetuity.
It is made available under a CC-BY 4.0 International license .

Supplemental Materials

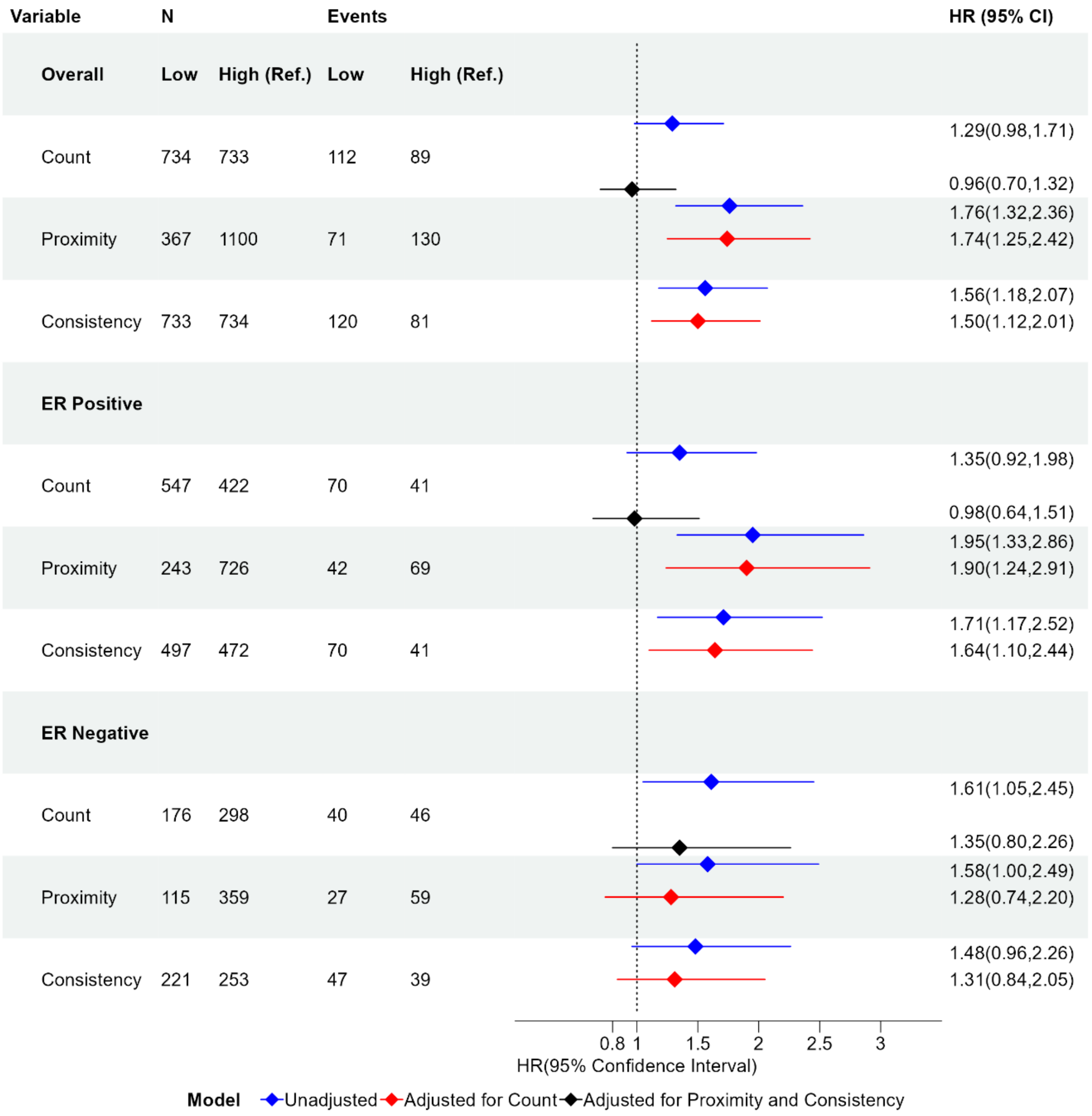

Figure 1: Results when using CD8 only and CD8 and FoxP3 double positive cells to compute proximity and consistency. Forest plot of hazard ratios and 95% confidence intervals for proximity, consistency, and lymphocyte count binary variables comparing low and high values when considering all participants and stratified by ER status. The Hazard ratio is given in the unadjusted case, adjusted for lymphocyte count, and adjusted for both proximity and consistency. The hazard ratio is with respect to the reference group (Ref.). The references groups are High Count, High Proximity, and High Consistency. Group size (N) and number of participants who experience recurrence (Event) are given for each group. HR: Hazard Ratio; 95% CI: 95% confidence Interval.

medRxiv preprint doi: https://doi.org/10.1101/2025.05.27.25328389; this version posted May 28, 2025. The copyright holder for this preprint (which was not certified by peer review) is the author/funder, who has granted medRxiv a license to display the preprint in perpetuity.
It is made available under a CC-BY 4.0 International license .

Models	Covariates	Estimated Hazard Ratio	Estimate p-value	p-value for Likelihood Ratio Test		
				Model 3	Model 4	Model 5
1	Low proximity	1.94	5.43e-06	0.016	0.88	
2	Low lymphocyte count	1.29	0.071		6.33e-05	1.57e-05
3	Low proximity<br>Low consistency	1.77<br>1.43	0.00015<br>0.017			0.55
4	Low lymphocyte count<br>Low proximity	0.98<br>1.96	0.88<br>5.11e-05			0.013
5	Low proximity<br>Low consistency<br>Low lymphocyte count	1.85<br>1.45<br>0.91	0.00025<br>0.014<br>0.55			

Table 1: Summary of multivariate cox proportional hazards models with proximity, consistency, and lymphocyte count binary covariates for all participants. Estimates for the covariate adjusted hazard ratios are provided as well as p-values for the significance of the hazard ratios. Models with nested covariates are compared using the likelihood ratio test and p-values for the significance of the complex model compared to the nested model are provided. The reference group for each of the covariates are high proximity, high consistency, and high lymphocyte count respectively.

QuPath Cell detection Tuning Parameters	
**Channel Used for Detection**	Hoechst
**Requested Pixel Size**	0.5
**Background Radius**	8
**Median Filter Radius**	0
**Sigma**	1.5
**Minimum Area**	10
**Maximum Area**	400
**Threshold**	11
**Cell Expansion**	3
**QuPath Classification Thresholds**	
**CD8 Positive Threshold**	25.12
**FoxP3 Positive Threshold**	80
**CK Positive Threshold**	31.62

Table 2: The tuning parameters used for cell detection and thresholds for cell classification in the QuPath functions.

medRxiv preprint doi: https://doi.org/10.1101/2025.05.27.25328389; this version posted May 28, 2025. The copyright holder for this preprint (which was not certified by peer review) is the author/funder, who has granted medRxiv a license to display the preprint in perpetuity.
It is made available under a CC-BY 4.0 International license .

		Training		Testing	
	**High (ref) vs. Low**	**HR**	**p-value**	**HR**	**p-value**
**overall**	Count	2.07	0.0001	1.65	0.0792
	Proximity	1.9	0.0003	2.02	0.0044
	Consistency	1.69	0.0101	1.06	0.8503
**ER Positive**	Count	3.19	3.35E-07	1.54	0.2393
	Proximity	2.58	0.0001	1.87	0.0709
	Consistency	2.3	0.0002	0.87	0.7256
**ER Negative**	Count	5.9	2.84E-06	1.46	0.307
	Proximity	7.59	0.0009	2	0.1888
	Consistency	5.63	0.001	2.8	0.0775

Table 3: Results from attempt to find optimal cutpoint for count, proximity, and consistency using a 2/3 to 1/3 training and test split in the overall dataset, the ER positive only, and the ER negative only. Hazard Ratios (HR) and p-values for the log-rank test are given for all cases in both the training and testing data. Results for testing data is based on the optimal cutpoint found using the training set.

The above table shows the results from optimizing the cutpoint for the high vs. low count, proximity, and consistency. We split the data based on a 2/3 to 1/3 training and testing split. Furthermore, we used stratified sampling on the recurrence data to ensure there would be an adequate number of participants with recurrence in the training and testing sets. We find an optimal cutpoint based on the quantile of the data which minimizes the p-value for the log-rank test. Though the cutpoints appear to perform well on the training set, in most cases there appears to be overfitting based on very different hazard ratios in the testing data. Furthermore, many results which were statistically significant in the training data are no longer statistically significant in the testing data.

medRxiv preprint doi: https://doi.org/10.1101/2025.05.27.25328389; this version posted May 28, 2025. The copyright holder for this preprint (which was not certified by peer review) is the author/funder, who has granted medRxiv a license to display the preprint in perpetuity.
It is made available under a CC-BY 4.0 International license .

Table 4: Cox Proportional Hazards Model with WSI TIL Scores

Covariates	Multivariate HR (95% CI)	Multivariate p-value	Univariate HR (95% CI)	Univariate p-value
**Low Proximity**	2.31 [1.25, 4,29]	0.01	1.94 [1.46, 2.58]	<0.005
**Low Consistency**	1.57 [0.91, 2.71]	0.1	1.62 [1.22, 2.15]	<0.005
**Low Count**	0.69 [0.36, 1.30]	0.25	1.29 [0.98, 1.71]	0.07
**Intratumoral Strength**	0.67 [0.36, 1.26]	0.22	0.48 [0.30, 0.78]	<0.005
**Intratumoral Forest**	1.01 [0.47, 2.16]	0.98	0.52 [0.29, 0.92]	0.03
**Intratumoral Desert**	0.60 [0.31, 1.15]	0.13	1.43 [0.84, 2.43]	0.18
**Peritumoral Strength**	0.66 [0.43, 1.02]	0.06	0.58 [0.42, 0.79]	<0.005

Table 5: Cox Proportional Hazards Model with Gene Expression based Immune Classes

Covariates	Multivariate HR (95% CI)	Multivariate p-value	Univariate HR (95% CI)	Univariate p-value
**Low Proximity**	1.88 [1.25, 2.84]	<0.005	1.94 [1.46, 2.58]	<0.005
**Low Consistency**	1.42 [0.98, 2.06]	0.06	1.62 [1.22, 2.15]	<0.005
**Low Count**	0.81 [0.53, 1.22]	0.31	1.29 [0.98, 1.71]	0.07
**CD8 T-Cell Signiture**	0.92 [0.78, 1.07]	0.28	0.84 [0.73,0.96]	0.01

Table 6: Cox Proportional Hazards Model with CD8 T-cell Signature

Covariates	Multivariate HR (95% CI)	Multivariate p-value	Univariate HR (95% CI)	Univariate p-value
**Low Proximity**	1.96 [1.31, 2.93]	<0.005	1.94 [1.46, 2.58]	<0.005
**Low Consistency**	1.45 [1.00, 2.10]	0.05	1.62 [1.22, 2.15]	<0.005
**Low Count**	0.84 [0.56, 1.27]	0.42	1.29 [0.98, 1.71]	0.07
**Quiet/Innate**	1.11 [0.73, 1.68]	0.63	1.35 [0.92, 1.96]	0.12
